# Prognostics and Clinical Outcomes in Patients Diagnosed With Acute Myeloid Leukemia (AML) in a Teaching Hospital

**DOI:** 10.7759/cureus.18915

**Published:** 2021-10-20

**Authors:** Hind A Alsulami, Maryam M Alnashri, Alanoud F Bawazir, Laila T Alrashid, Raghdah A Dly, Yusr A Alharbi, Mohamad H Qari

**Affiliations:** 1 Hematology Department, King Abdulaziz University Hospital, Jeddah, SAU

**Keywords:** teaching hospital, bone marrow biopsy (bmb), clinical outcome, prognosis, acute myeloid leukemia (aml)

## Abstract

Background: Acute myeloid leukemia (AML) is a heterogeneous disease. Prognosis and survival depend on several factors that determine tumor behavior and response to therapy. AML has a poor prognosis that depends on several factors: patient's age, gender, body mass index (BMI), baseline white blood cells count, and bone marrow blast (BMB) cell count at the time of diagnosis. Therefore, this study aimed to determine the prognostic role of these factors and their impact on outcomes, and how these prognostic factors may affect AML patients before and after induction chemotherapy.

Methods: The study design is an observational, retrospective record review. We included records of patients diagnosed with primary and secondary AML who received chemotherapy between 2013 and 2019 at King Abdulaziz University in Jeddah, Saudi Arabia. Data were extracted from medical records, entered into an Excel sheet (Microsoft Corp., Redmond, WA), and analyzed using SPSS Statistics, version 25 (IBM Corp., Armonk, NY).

Results: Forty-two AML patients who were started on chemotherapy were analyzed. The mean age at diagnosis was 35 ± 22.2 years; 52.4% were male. The ability to achieve the first remission varied according to age group; the 21-45 age group had the higher ability and survival rate of 75.0%*. *On the other hand, the mortality incidence was higher (at 70.0%) in both the 11-20 and the 46-70 age groups. A strong negative correlation was observed between age and survival duration after treatment (SDAT) (r = - 0.618, p = 0.004). The death incidence was increased in the BMI ranges that were under and above the normal weight range. SDAT differed significantly between the three groups in favor of the normal-weight patients (p = 0.019). We found that patients with BMB < 5 had the most deaths. There was a significant negative association between BMB and days to achieve the first remission after treatment (p = 0.033).

Conclusion: Age, BMI, and BMB are considered effective prognostic factors for AML patients.

## Introduction

Acute myeloid leukemia (AML) is a malignant hematological disease characterized by the infiltration of the bone marrow, blood, and other tissues through clonal expansion and poorly differentiated cells of the hematopoietic system [1]. AML accounts for the largest number of deaths in leukemia patients in the United States [1]. It affects bone marrow (BM) stem cells, specifically, the myeloid cells resulting in a higher increase in myeloid blasts number (the immature form) and arresting the maturation of these blasts [2,3]. These nonfunctional cells will cause hematopoietic dysfunction resulting in potential decreases in other blood cells, including red blood cells (RBCs), white blood cells (WBCs), platelets (PLTs) [2,3].

According to the WHO, detecting more than 20% of BM cells as myeloid blasts is diagnostic for AML [3]. The real causes of AML have not been identified explicitly so far. According to the American Cancer Society, there are many risk factors for AML, including age (mainly in adults) and sex (it is the third most common malignancy affecting males and the fifth in females) [4]. In addition, other factors include smoking, chemotherapy exposure for other malignancy treatment, being exposed to certain chemicals or radiation, and having a genetic syndrome or mutation [5,6]. The typical treatment for most types of AML is chemotherapy, sometimes combined with a targeted therapy drug [6]. This might be followed by a stem cell transplant, surgery, or radiation therapy [6]. 

Several variables have been reported as prognostic factors, including age, sex, and body mass index (BMI) with different clinical outcomes [7-10]. The backbone therapy for AML is intensive chemotherapy regimens such as cytarabine and an anthracycline ("7 + 3" regimen), with a complete remission average of 60%-80% in younger adults and decreased efficiency in the elderly who are above 60 years old, with full response rate equal to 40%-60% in the United States [11]. Although there is no clear dividing line when considering age in AML, in most studies, "older adults" were defined as over 60 years [12]. AML was incurable fifty years ago [13]. Recently, a study conducted on adult AML patients showed that approximately 35%-40% of patients aged 60 years or younger and 5%-15% of patients older than 60 years had been cured [13]. A study conducted in Iran covered 96 patients suggested that the age at diagnosis was considered as a significant prognostic factor with patients 35 years old or younger showing a survival rate of 43.9% and patients older than 35 years, 36.3%; males demonstrated a higher survival rate than females [2].

In addition, patients with WBC count < 20 × 10^3/μL had a survival rate of 49.1%, while those with WBCs > 20 × 10^3/μL had a survival rate of 25.6% [2]. Moreover, another study estimated WBCs and platelets as prognostic factors and concluded that WBCs more than 50 × 10^9/L reflected shorter relapse-free survival, higher relapse rate, and shorter overall survival, while the lower WBC count showed longer relapse-free survival [14]. The PLT count < 100 × 10^9/L and ≥ 100 × 10^9/L was not statistically significant regarding the frequency of relapse and overall survival [14]. In addition, the value of the WBC index (WBC index = WBC [% of marrow blasts/100]) has been investigated in France using the following three-group classification (low WBC index, 2.5; intermediate WBC index, 2.5-20; high WBC index, 20 or more); after three years, the estimated overall survival was 74% in patients with low WBC index, 66%in patients with intermediate WBC index, and 47% in patients with high WBC index [15]. Bone marrow blast (BMB) count (immature nonfunctional cells) and the peripheral blood blast (PBB) count percentages have been compared in four diseases (AML, myeloid dysplastic syndromes, acute lymphoblastic leukemia, chronic myeloid leukemia), and it was found that BMB count was high in all groups, with an exception for chronic myeloid leukemia that was found to have a higher percentage of peripheral blood blasts count [16].

However, per our knowledge, studies that consider and explore the prognostic role of blood blasts in AML patients explicitly have not emerged yet. The genetic aspect plays a significant role in the susceptibility and prognosis of the disease [17]. According to the cancer report, AML represented 6.9% of all adult cancers in Saudi Arabia in 2009 [3]. However, there are limited studies and findings related to the prognosis of AML patients in Saudi Arabia. Understanding such concepts might be helpful by indicating the prognosis of the disease, the efficacy of treatment, and even the type of treatment that should be used. Our study aimed to investigate AML prognostic factors including age, sex, BMI, RBCs, WBCs, and PLT count, in addition to BMB and PBB factors, and to evaluate the chemotherapy response of patients in King Abdulaziz University Hospital (KAUH), Jeddah, Saudi Araba.

## Materials and methods

This study is a retrospective record review which was conducted by reviewing all AML patients' records and including those who were on chemotherapy from 2013 to 2019, at King Abdulaziz University Hospital, Jeddah, Saudi Arabia. It was approved by the Unit of Biomedical Ethics at King Abdulaziz University Faculty of Medicine (Reference No. 439-21). Demographic data were scanned (sex, age, BMI, and blood type), the examined prognostics were baseline white blood cells (WBCs), red blood cells (RBCs), platelets (PLTs), peripheral blood blast (PBB), bone marrow blast (BMB) counts in addition to age and BMI. The data were correlated with the following outcomes: an initial remission after chemotherapy induction, the duration required to achieve it, mortality, and the survival duration after treatment (SDAT) until the patient's death or the end of study follow-up. The following normal laboratory values were used as reference for the tested parameters: BMI, 18.5-24.9 kg/m^2; WBC, 4.5-11 × 10^9/L; PBB, 0%; BMB, ≤ 5%; RBC, 4-6 million cells/µL; platelets, 150-450 × 10^3 platelets/µL.** **Data were entered into an Excel sheet (Microsoft Corp., Redmond, WA) and analyzed using SPSS Statistics, version 25 (IBM Corp., Armonk, NY). The statistical analyses used for univariate qualitative and quantitative data were frequency and measure of central tendencies (MCTs), respectively. We used the following tests for bivariate qualitatives and/or normally-distributed quantitative comparison: Chi-square test, independent samples t-test, one-way analysis of variance (ANOVA), and Pearson correlation. Mann Whitney U test, Kruskal Wallis H test, and Spearman correlation were used to test non-normally distributed quantitative (survival duration after treatment, RBC, and platelets count) and qualitative data. P-value < 0.05 was considered significant.

## Results

Forty-six patients were diagnosed with AML and had chemotherapy induction in the hospital from 2013 to 2019. Four were excluded due to incomplete data regarding chemotherapy induction or not completing the follow-up at the hospital. Thus, 42 patients were analyzed to explore the prognostics and clinical outcomes.

The population size and percentage

The mean age at diagnosis was 35 ± 22 years old. Twenty-two patients were male (52.4%). The most frequent blood types were O+ (16, 39%) and A+ (13, 31.7%). Reaching the first remission after chemotherapy induction was achieved by 22 patients (52.4%) with an elapsed duration average of 74 ± 49.6 days; 54.5% of these patients were still alive. Of the total 42 patients, 23 (54.8%) died, but the date of death was not documented for four of them, and thus, the survival duration after treatment (SDAT) was accounted for the remaining 19 patients. The median SDAT was 26 days, IQR = 194, with a minimum of 0 days and a maximum of 901 days. Nineteen (45.2%) patients were still alive at the end of the study (13th April 2019), with a mean survival of 946 days. Regarding AML types and survival, 28 (66.7%) had primary AML, including two (7.1%) who had experienced previous chemotherapy, and 16 (57%) were still alive. Of the 14 patients (33.3%) who had secondary AML, three (21.4%) have survived (P = 0.028).

BMI at diagnosis

The death incidence was increased in the BMI ranges that were under and above the normal range. Of the total deaths, six patients had underweight BMI, seven patients had normal BMI, and 10 patients had overweight BMI, but the difference was not significant (P = 0.110). The SDAT was significantly different between the three groups favorably to the normal-weight patients (P = 0.019); the median was 446 days for underweight (n=3), 27 days for normal weight (n=7), and 18 days for overweight (n=9).

Age at diagnosis

The ability to reach the first remission has been found to be varied among age groups. Patients aged 21-45 years showed the best ability to get the remission. Regarding mortality, the incidence was higher (at 70.0%) in both the 11-20 and 46-70 age groups, while survival was higher in the 21-45 group at 75.0% (Table 1). Moreover, a strong negative correlation was observed between age and the SDAT (r = -0.618, P = 0.004).

**Table 1:** Prognostic factors prediction in AML patients N = total number.

Prognostic Factor at Diagnosis, N	Mortality N (%)	P-value
Body Mass Index, 42		
Normal weight, 18	7 (38.9%)	0.110
Overweight, 13	10 (76.9%)	0.110
Underweight, 11	6 (54.5%)	0.110
Age groups, 42		
(0 – 10), 6	3 (50.0%)	0.142
(11 – 20), 10	7 (70.0%)	0.142
(21 – 45), 12	3 (25.0%)	0.142
(46 – 70), 10	7 (70.0%)	0.142
(> 70), 4	3 (75.0%)	0.142
Wight Blood Cells, 42		
Normal, 4	2 (50.0%)	0.726
High, 27	16 (59.3%)	0.726
Low, 11	5 (45.5%)	0.726
Bone Marrow Blast, 33		
(5 – 19%), 8	4 (50%)	0.169
(< 5%), 7	6 (85.7%)	0.169
(>= 20%), 18	8 (44.4%)	0.169
Peripheral Blood Blast, 39		
(< 20%), 8	3 (37.5%)	0.163
(>= 20%), 31	20 (64.5%)	0.163

WBC at diagnosis

We noted that the death rate tended to be increased in patients with high WBC (P = 0.726). 

BMB at diagnosis

Out of 42 patients, nine patients' data was missed. We found patients with BMB < 5% had the highest death events, and the incidence decreased as the BMB increased (Table 1). There was a significant adverse relationship between BMB and the days elapsed until reaching the initial remission after treatment; the longest duration was observed in BMB < 5% with an average of 134 days, 95% CI (83 to 185.6), (P = 0.033). 

PBB at diagnosis

Out of 39 patients, it was found that as PBB increased at diagnosis, so did the death events. This trend was opposite to the one seen for BMB at diagnosis.

The results did not show a notable prognostic trend for RBCs and platelet count, although the median at diagnosis date was low (3, 51), respectively.

## Discussion

A comprehensive understanding of AML is required to treat patients appropriately. Consequently, we evaluated the role of several prognostics with certain outcomes spontaneously in the same population since the disease is influenced by the genome and the genetic expression status and might vary in different regions or cultures [18].

Body mass index is a number that reflects the body weight-adjusted to height. Normal values are 18.5-24.9 kg/m^2. while 25-29.9 kg/m^2 are considered overweight [19]. Recent studies have marked out the effect of BMI on the outcome of AML patients’ treatment. A study reported that children with normal body weight have better outcomes compared with overweight and underweight patients, which confirms our findings [19]. Although multiple reports show the relationships between adult AML patients’ body mass index and the clinical outcomes, they only studied overweight status [20-23]; other studies showed that underweight patients get poorer outcomes compared with normal-weight patients [24]. In the present study, underweight and overweight patients showed poorer clinical outcomes; they had higher death incidence compared with normal-weight patients. 

As is known about the age prognosis effect, aging is a poor prognostic from several aspects [7]. The finding suggests that as patients get older, they will experience an unfavorable prognosis since the survival duration after treatment decreases; patients aged 65 years or older have a dismal prognosis even after intensive chemotherapy, irrespective of their cytogenetic risk [25]. Moreover, younger patients reached the first remission at a higher percentage than older patients. This may be because of the increase of chromosomal abnormalities associated with the disease as a person gets older [7]. Older patients may additionally have other health problems that make it difficult for them to deal with the side effects of treatments for AML. 

Our results indicate that there is a trend toward a poor prognostic role for WBC. As WBC increases, from a WBC interval to another higher one, the death rate increases by approximately 6%. It is not significant, but a study in Iran supported the assumption [2]. However, a study in France found no significant impact of WBC count on reaching complete remission (CR); the WBC index as a prognostic factor (WBC*BMB/100) showed a strong negative prognosis for overall survival and disease-free survival, p-value = 0.002 [15]. We suggest that these differences between the studies, especially between previous studies and ours are due to the different populations and the small sample size compared with the other studies (42 vs. 96 and 154, respectively) [2,15]. 

Our results suggest that the increased BMB count mainly has a negative effect on survival. On the one hand, BMB was not a parameter of concern at diagnosis, as many of our patients were diagnosed with AML without considering bone marrow aspiration. On the other hand, almost all our patients had documented blood film for PBB count at diagnosis, which showed that a PBB of more than 20% was associated with higher mortality, reflecting its importance in disease progression. However, our finding supports a study comparing BMB and PBB in the evaluation of AML where the authors found that both BMB and PBB can be used for diagnosis and mainly PBB for monitoring AML patients [26]. In addition, there was an unobvious impact for RBCs and platelets. We did not find a study to compare our result for RBC and platelets, which did not show any notable role.

Several factors limited the study. Though the study involved all AML patients in the hospital between 2013 and 2019, the sample size was relatively small, and the documentation was incomplete in some cases. Also, the absence of measurements of certain variables held us from conducting the study more comprehensively. For those factors (platelets, RBC, and sex) that did not show significant influence, a study with a higher sample size is recommended to confirm their impact on our population. It should be in multicenter to reach a larger population that involves most existing races and conditions.

## Conclusions

AML is considered a devastating disease characterized by a variety of recurring gene mutations. This study aimed to check how prognostic factors affect patients with AML before and after chemotherapy induction. We found that age, BMI, BMB were the more effective prognostic factors, followed by PBB and then WBCs. Our data indicated that BMB and PBB have inverse roles with mortality. It is suggested that patients' age at diagnosis is the strongest patient‐related prognostic factor. However, we didn't find any significant impact of WBCs on AML patients. To gain new insights for future studies, we recommend other authors consider more laboratory data such as cytogenetic and gene mutation data.
